# Gene conversion yields novel gene combinations in paralogs of GOT1 in the copepod *Tigriopus californicus*

**DOI:** 10.1186/1471-2148-13-148

**Published:** 2013-07-12

**Authors:** Christopher S Willett

**Affiliations:** 1 Department of Biology, University of North Carolina, Chapel Hill, NC 27599-3280, USA

**Keywords:** Gene duplication, Concerted evolution, Cytoplasmic GOT, Aspartate transaminase, *Tigriopus californicus*

## Abstract

**Background:**

Gene conversion of duplicated genes can slow the divergence of paralogous copies over time but can also result in other interesting evolutionary patterns. Islands of genetic divergence that persist in the face of gene conversion can point to gene regions undergoing selection for new functions. Novel combinations of genetic variation that differ greatly from the original sequence can result from the transfer of genetic variation between paralogous genes by rare gene conversion events. Genetically divergent populations of the copepod *Tigriopus californicus* provide an excellent model to look at the patterns of divergence among paralogs across multiple independent evolutionary lineages.

**Results:**

In this study the evolution of a set of paralogous genes encoding putative aspartate transaminase proteins (called GOT1 here) are examined in populations of the copepod *T*. *californicus*. One pair of duplicated genes, *GOT1p1* and *GOT1p2*, has regions of high divergence between the copies in the face of apparent on-going gene conversion. The *GOT1p2* gene also has unique haplotypes in two populations that appear to have resulted from a transfer of genetic variation via inter-paralog gene conversion. A second pair of duplicated genes *GOT1Sr* and *GOT1Sd* also shows evidence of gene conversion, but this gene conversion does not appear to have maintained each as a functional copy in all populations.

**Conclusions:**

The patterns of conservation and sequence divergence across this set of paralogous genes among populations of *T*. *californicus* suggest that some interesting evolutionary patterns are occurring at these loci. The results for the *GOT1p1*/*GOT1p2* paralogs illustrate how gene conversion can factor in the creation of a mosaic pattern of regions of high divergence and low divergence. When coupled with rare gene conversion events of divergent regions, this pattern can result in the formation of novel proteins differing substantially from either original protein. The evolutionary patterns across these paralogs show how gene conversion can both constrain and facilitate diversification of genetic sequences.

## Background

Gene conversion can impact the evolution of duplicated genes in a number of different ways including both impeding sequence divergence between genes and transferring variation between them [1]. Gene conversion is a common mechanism of unidirectional homologous recombination in eukaryotes that results in a cut-and-paste like copying of sequence between similar alleles that are either at the same locus or at another locus in the same genome (reviewed in Chen et al. [2]). Concerted evolution can result from loci undergoing repeated gene conversion, which causes duplicated genes to evolve in tandem and not diverge from one another over evolutionary time. Not all duplicated genes are subject to gene conversion, in fact, surveys in mammals and fruit flies suggest that only about ten percent of paralogous copies show signs of gene conversion, and only a small fraction of the total sequence length is typically impacted [3,4].

Duplicated genes that are experiencing concerted evolution typically will go through a series of phases of differential divergence. Rates of gene conversion between sequences go down as the sequences become more dissimilar. For gene duplicates undergoing some level of concerted evolution, divergence between them will not begin to increase markedly until a threshold of sequence divergence is breached (as high as 20 percent [5]). Models of this process suggest that there will typically be a long period of evolution with only low levels of divergence until a threshold level of divergence is passed at which point the rate of divergence will increase [6]. Selective divergence can counter this homogenization and lead to the establishment and maintenance of regions of higher sequence divergence in the face of gene conversion if differences in specific regions of the gene between the two duplicates are adaptive (e.g. with neofunctionalization [7]). Teshima and Innan [7] propose scanning for this specific pattern as a method of identifying such regions undergoing selection. Using this method in a study in yeast, Takuno and Innan [8] identified two sets of duplicated heat shock proteins that likely fit this model.

In addition to the role outlined above in slowing or countering adaptive divergence between duplicated genes, gene conversion can also play a role in transferring adaptive variation between duplicate genes. Under such a scenario, gene conversion acts to increase the effective population size of the duplicated genes, making selection more efficient. This transfer can spread advantageous variation and remove deleterious mutations [9,10]. A number of studies have shown that gene conversion between duplicate genes with some degree of initial divergence between them can result in the introduction of high levels of variation at the converted locus [11-18]. For many of these cases, this variation appears to be adaptive with a number of these genes under selection for higher haplotype diversity (e.g. MHC, attacin, and resistance genes in plants).

The copepod *Tigriopus californicus* has a set of unique features that makes it useful system in which to look at patterns of molecular evolution in duplicated genes. *T*. *californicus* exists in a series of extensively genetically divergent populations that have undergone substantial periods of independent evolution from one another. This species occurs in rocky upper intertidal pools along the Pacific coast of North America from Mexico to Alaska. Populations of this species can be highly genetically divergent from one another even over relatively short distances, with mitochondrial DNA (mtDNA) divergences greater than 20 percent between populations [19-21]. Divergence in the nuclear genome is lower but still substantial, likely reflecting a substantially higher rate of mutation for the mtDNA [22]. Even with these higher rates of mtDNA evolution, the levels of divergence among populations suggest that these populations have been evolving fairly independently of one another for long periods of time. Genomic resources are being developed for this species and now include published transcriptomes from a pair of populations, and these resources facilitate the characterization of paralogs [23].

In this paper the molecular evolution of a set of aspartate transaminase-encoding homologs is examined in populations of *T*. *californicus*. A putatively mitochondrially targeted homolog was previously identified from this species [24] and named after the corresponding allozyme locus (*GOT2*, the enzyme aspartate transaminase was formerly called glutamate-oxaloacetate transamine; EC 2.6.1.1). Five additional homologs are described in this paper that have originated from a series of gene duplication events in the evolutionary lineage leading to this species. Sequence similarity suggests that these genes are likely to be cytoplasmically targeted GOT1 proteins. Two sets of somewhat more recently duplicated pairs of genes show strong evidence of gene conversion. In this paper the differential impact of gene conversion on the evolution of these two pairs of duplicated genes is examined.

## Results

### Identification of GOT paralogs

Five new paralogous genes were identified from a PCR-based screen of expressed sequences from the copepod *T*. *californicus* that are homologous to genes encoding aspartate transaminase proteins (in addition to the previously identified *GOT2* gene [24]). These genes were sequenced in four populations including three from southern California, San Diego (SD), La Jolla (LJS), and Abalone Cove (AB), and one from central California, Santa Cruz (SCN). Four of these homologs, GOT1p1/GOT1p2 and GOT1Sd/GOT1Sr, had moderate levels of genetic divergence within pairs (Table 1) but high levels of divergence between pairs (43 percent amino acid identity). These two pairs are also highly divergent from the paralog GOT1_6a, with 41 percent and 39 percent amino acid identity between GOT1_6a and the GOT1p1 and GOT1Sr proteins respectively. The GOT1p1/GOT1p2 proteins appear to be orthologous to other arthropod cytoplasmic GOT1 proteins (58 percent amino acid identity with *Drosophila melanogaster* GOT1 isoform A). Phylogenetic analyses clearly place the GOT1p1/GOT1p2 paralogs with other arthropod cytoplasmic GOT1 proteins and confirm the close relationship of the GOT1Sd/GOT1Sr proteins but do not consistently resolve the relationships of these two proteins and GOT1_6a with other organisms’ homologs (Additional file 1: Figure S1). These three proteins fall basal to the other GOT1 homologs from animals, but this placement in not strongly supported in either Bayesian or parsimony phylogenetic analyses.

**Table 1:** **Fixed genetic divergence in coding regions for orthologs and paralogs of GOT1 in populations of *T***. ***californicus***

	**Between GOT1p1**/**GOT1p2 paralogs**				**GOT1p1 orthologs**			**GOT1p2 orthologs**		
	SD p1/SD p2	LJS p1/LJS p2	AB p1/AB p2	SCN p1/SCN p2	SD p1/LJS p1	SD p1/AB p1	SD p1/SCN p1	SD p2/LJS p2	SD p2/AB p2	SD p2/SCN p2
k_s_	0.237	0.245	0.354	0.354	0	0.046	0.082	0	0.050	0.062
k_a_	0.025	0.024	0.032	0.033	0	0.002	0.003	0	0.006	0.003
k_a_/k_s_	0.105	0.100	0.091	0.094	-	0.048	0.040	-	0.111	0.053
	Between GOT1Sd/GOT1Sr paralogs				GOT1Sd orthologs			GOT1Sr orthologs		
	SD Sd/SD Sr	LJS Sd/LJS Sr	AB Sd/AB Sr	SCN Sd/SCN Sr	SD Sd/LJS Sd	SD Sd/AB Sd	SD Sd/SCN Sd	SD Sr/LJS Sr	SD Sr/AB Sr	SD Sr/SCN Sr
k_s_	0.034	0.016	0.009	0.036	0.008	0.010	0.048	0.004	0.027	0.052
k_a_	0.010	0.002	0.015	0.016	0.003	0.018	0.020	0.002	0.017	0.018
k_a_/k_s_	0.307	0.155	1.557	0.461	0.311	1.845	0.419	0.624	0.653	0.354
	GOT1_6a orthologs									
	SD 6a/LJS 6a	SD 6a/AB 6a	SD 6a/SCN 6a	LJS 6a/AB 6a	LJS 6a/SCN 6a	AB 6a/SCN 6a				
k_s_	0.004	0.032	0.040	0.045	0.058	0.028				
k_a_	0.001	0.002	0.011	0.004	0.013	0.011				
k_a_/k_s_	0.316	0.077	0.279	0.084	0.219	0.399				

Levels of divergence in coding region are calculated for fixed differences (excluding polymorphism) for k_s_ (synonymous substitutions per site) and k_a_ (non-synonymous substitutions per site) with a Jukes-Cantor correction. Raw numbers of changes and sites can be found in Additional file 2: Table S1. Note that the *GOT1Sd* sequences spanned only one-half of the coding region of *GOT1Sr* (504 bp vs 1122 bp) and that the reading frame was corrected when calculating numbers of synonymous and nonsynonymous substitutions.

In addition to the large amount of amino acid divergence among the more divergent GOT1 paralogs, there are also a number of structural differences at these loci. The *GOT1*_*6a* gene and the *GOT1Sr* genes each have four introns in the same positions in the gene (as assessed by their position in the amino acid alignment). *GOT2* also has four introns but only one of these shares a position with those of the *GOT1*_*6a* and *GOT1Sr* genes (the third intron). The size of this third intron varies widely from 152 bp in *GOT1*_*6a* to 3894 bp for the SCN population for *GOT1Sr* (the other three populations each have a 2723 bp for this intron in the *GOT1Sr* gene). Interestingly, the *GOT1p1*/*GOT1p2* genes lack introns completely. The transcript for each of these genes is between 1257 bp for *GOT1Sr* and 1532 bp for *GOT1*_*6a*, while the coding regions are all close to 1224 bp (with *GOT1*_*6a* being 1233 bp). We did not obtain sequence corresponding to the first 618 bp of the coding region for the *GOT1Sd* gene, but the sequenced portion is consistent with the presence of the final three introns. For the AB and LJS populations the second and third introns respectively have polymorphisms that would alter the predicted splice sequences for the *GOT1Sd* gene.

Five of these six GOT genes can be found in the published transcriptome dataset derived from the SD and SCN populations of *T*. *californicus*[23] with only the *GOT1Sd* gene missing. These data also give some hints as to the relative expression levels of these genes. Total read numbers per gene are somewhat low overall in this 454 dataset but the highest counts were found for the *GOT2* gene with 247 reads and the *GOT1p1*/*GOT1p2* genes with 154 reads summed over both copies. Examination of the proportion of reads from the diagnostic regions of the *GOT1p1*/*GOT1p2* genes suggests that the expression of the *GOT1p1* gene is about 6-fold higher than that of the *GOT1p2* gene. The *GOT1*_*6a* and *GOT1Sr* genes had fewer than 10 reads each suggesting that they are expressed at a much lower level. Consistent with its absence from the transcriptomes, our lab found no expression of the *GOT1Sd* gene using qualitative RT-PCR assays in the San Diego (SD) population, but we did find expression of both the *GOT1Sr* and *GOT1p1*/*GOT1p2* paralogs (Willett CS, unpublished data). Sequences of mRNA obtained from individual copepods from these experiments were identical to haplotypes obtained via direct sequencing from the coding regions.

### Divergence in GOT1 paralogs and gene conversion

**Figure 1:**
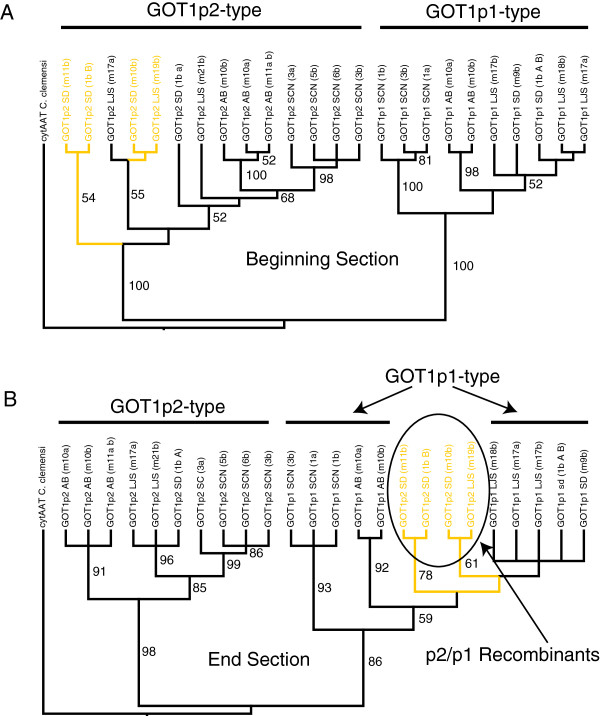
**Phylogenetic relationships amongst the *T*. *californicus GOT1p1*/*GOT1p2 *paralogs.** The tree is the 50% majority rule consensus tree of 16 most parsimonious trees obtained by using either **(A)** the first portion of the gene (1–923 bp) or **(B)** the second portion of the gene (924–1206). Numbers on the branches give the boostrap values obtained from 10 000 replicate bootstrap searches using the parsimony criterion. The tree was rooted using the putative ortholog ACO15246 cytAAT from *Caligus clemensi*. The *GOT1p2* haplotypes in the SD and LJS populations that appear to have been converted by the *GOT1p1* sequence in the end section are highlighted.

The duplication events that produced the *GOT1p1* and *GOT1p2* paralogs and the *GOT1Sd* and *GOT1Sr* paralogs appear to have occurred in the *Tigriopus* lineage prior to the split of the four populations examined in this study. This can be seen for the *GOT1p1* and *GOT1p2* paralogs in the higher levels of divergence between paralogs within a population as compared to the divergence between presumed orthologs between populations (Table 1) and from phylogenetic analyses (Figure 1A). In both of the *GOT1p1* and *GOT1p2* paralogs there are more synonymous substitutions than nonsynonymous substitutions between orthologous copies across populations and this is reflected in the low values of K_a_/K_s_ (with *GOT1p1* showing a higher degree of conservation). Comparisons across paralogous copies within populations show the same pattern of relatively low K_a_/K_s_ values (Additional file 2: Table S1). The number of nonsynonymous substitutions is higher across populations for the *GOT1Sd* and *GOT1Sr* paralogs with correspondingly higher K_a_/K_s_ ratios (with some exceeding one; Table 1). The *GOT1Sd* and *GOT1Sr* paralogs have not diverged substantially in the sequenced coding regions, but the third intron has diverged to the degree that much of it cannot be aligned between the paralogs (it also differs significantly in size-761 bp for *GOT1Sd* and 2723 bp for *GOT1Sr* in the SD population).

**Figure 2:**
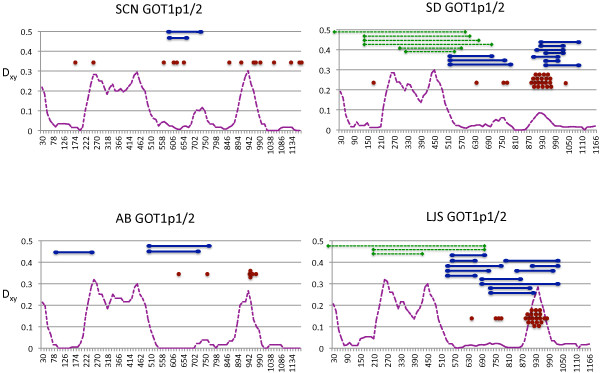
**Variation in levels of divergence between *T*. *californicus GOT1p1*/*GOT1p2 *paralogs and regions of predicted gene conversion.** Divergence (D_xy_) between the *GOT1p1* and the *GOT1p2* paralogs is calculated over a sliding window with a window size of 60bp and a step size of 12bp. Gene conversion events predicted by the program GENECONV are shown as lines above the divergence plots with dashed lines indicating intra-paralog and solid lines indicating inter-paralog gene conversion events. The dots indicate another related signature of gene conversion, sites that are shared between paralogs within a population (either polymorphic or fixed) but not across populations. Results are shown separately for each of four *T*. *californicus* populations (SCN, SD, AB, LJS). The full set of predicted regions of gene conversion are given in Additional file 3: Table S2 with associated statistics from GENECONV.

**Figure 3:**
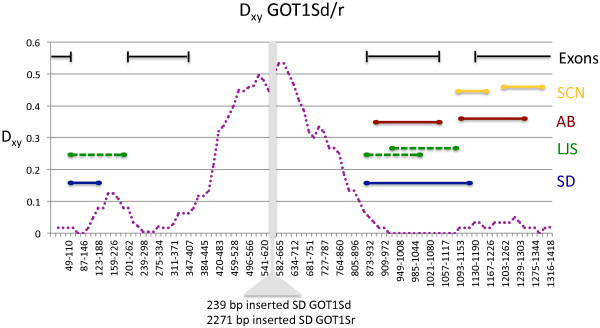
**Levels of divergence between *GOT1Sd*/*GOT1Sr *paralogs from *T*. *californicus *with regions of predicted gene conversion.** Divergence (D_xy_) is calculated across all four populations between the two paralogs of *GOT1S* with a sliding window of size 50 bp and 10 bp steps. Patterns of divergence were very similar across populations with the slight exception of the SCN population comparison that showed a small peak (0.15) of divergence centered over the 985–1044 window. Only inter-paralog conversion events are given for these two paralogs with population identity shown on the righthand side of the figure. Exonic regions are indicated at the top of the figure. Note that the central intron varies in size between *GOT1Sd* and *GOT1Sr* and cannot be aligned. The full set of predicted regions of gene conversion are given in Additional file 3: Table S2 with associated statistics from GENECONV.

**Figure 4:**
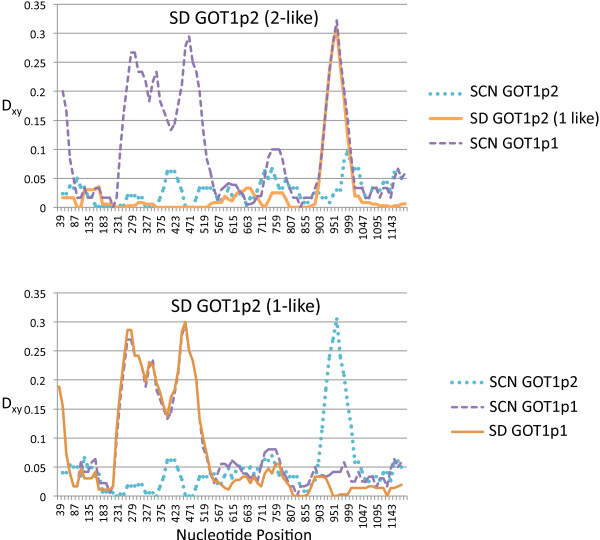
**Apparent transfer of variation via inter-paralog gene conversion for *GOT1p1 *and *GOT1p2*.** The transfer of variation from *GOT1p1* to *GOT1p2* for certain alleles is evident in the patterns of divergence across each gene in comparisons of SCN and SD paralogs. In the top graph comparisons are done between the SD *GOT1p2* (2-like) paralog and the SD *GOT1p2* (1-like), and SCN paralogs. In the bottom graph comparisons are made between the *GOT1p2* (1-like) paralog and the SD *GOT1p1* paralog, and SCN paralogs. The *GOT1p2* (1-like) alleles appear to have been converted by the *GOT1p1* copy for the peak near position 951 because they are divergent from the GOT1p2 (and 2-like) alleles but similar to the GOTp1 copies. In contrast, the *GOT1p2* (2-like) alleles have no large peaks of divergence with the GOT1p2 copies from the SCN population (similar patterns are seen for comparisons with the AB and LJS paralogs).

It appears that a history of past and on-going gene conversion events has left a strong impression on the patterns of genetic variation within and among paralogs of *GOT1* in *T*. *californicus*. Using the program GENECONV [25] a series of inter- and intra paralog conversion events are evident (Figures 2 and 3; Additional file 3: Table S2). If we count non-overlapping predicted gene conversion events as a minimum number of gene conversion events, there have been at least two inter-paralog and one intra-paralog gene conversion events between *GOT1p1* and *GOT1p2* genes for SD and LJS populations (with the intra-paralog events occurring between alleles of *GOT1p2*). A minimum of two inter-paralog gene conversion events are also predicted for the AB population and one for the SCN population with no intra-paralog events for either population (predicted conversion events are listed in Additional file 3: Table S2). For the *GOT1Sd* and *GOT1Sr* paralogs there are a minimum of two inter-paralog gene conversion events predicted for the SD, LJS, and AB populations and one for the SCN population. For this pair, intra-paralog gene conversion events are limited to the GOT1Sd paralog, and there are at a minimum two of these for the SCN population and one for the SD and LJS populations. Also, for the *GOT1Sd* and *GOT1Sr* paralogs, the inter-paralog gene conversion events are largely restricted to the exons (with the third intron too divergent in sequence to align over much of its length as mentioned previously). The predicted intra-paralog gene conversion events for *GOT1Sd* are all predicted to occur in this same intron (Additional file 3: Table S2). In contrast, for the *GOT1p1* and *GOT1p2* paralogs, there are no introns in the sequenced region of the gene, and the inter-paralog gene conversion events overlap primarily with the regions of low genetic divergence between paralogs. Comparisons of *GOT1* homologs from a range of arthropods and two vertebrates suggest that conserved amino acid regions are scattered across the protein and not centered only in the regions with evidence for gene conversion events (Additional file 4: Figure S2). Even for the regions with no genetic divergence between *GOT1p1* and *GOT1p2* paralogs from the same population, there is still some divergence among populations for each ortholog (Figure 4).

### Polymorphism capture via gene conversion

The SD and LJS *GOT1p1* and *GOT1p2* paralogs stand out for their elevated levels of polymorphism in comparison to other genes in these populations (Table 2; [22,26]), and it appears that this pattern could stem from inter-paralog gene conversion events introducing variation. For these genes levels of synonymous polymorphism are nearly an order of magnitude higher than levels from other genes in these *T*. *californicus* populations, which had an average π value of 0.003 for SD and 0.006 for LJS across a set of eight nuclear-encoded genes [22,26]. The *GOT1p2* genes in the SD and LJS populations also have a large number of nonsynonymous polymorphisms for the *GOT1p2* paralog in addition to synonymous polymorphisms. For the peak of divergence at position 930 (Figure 2) alleles can differ by as many as eight nonsynonymous polymorphisms and fourteen synonymous polymorphisms. It appears that many of these polymorphisms are the result of gene conversion introducing variation from the *GOT1p1* copy to the *GOT1p2* copy as can be seen by examining patterns of divergence between alleles (Figure 4). Phylogenetic analyses of each end of the gene also confirm this pattern of differential history for these SD and LJS *GOT1p2* haplotypes (Figure 1). Inspection of all of the sequences also suggests a number of other candidate SNPs that are likely to result from such inter-paralog events in these two populations and in the other two populations as well (dots in Figure 2).

**Table 2:** **Levels of polymorphism in GOT paralogs in *T***. ***californicus***

**Pop.**	**Paralog**	**# Hap.**	**π**_**syn**_	**S**_**syn**_	**π**_**nonsyn**_	**S**_**nonsyn**_	**π**_**syn+nc**_	**S**_**syn+nc**_	**Taj. D**	**Indels in coding region**
SD	GOT1p1	6	0.06674	3	0	0	NA	NA	1.09	
	GOT1p2	10	0.04501	34	0.00675*	15	0.04359	34	0.21	70 bp insert (one haplotype)
	GOT1Sr	4	0	0	0	0	0.00076	5	0.372	
	GOT1Sd	10	0	0	0.00538*	5	0.00466	12	1.144	1bp fixed
	GOT1_6a	10	0.00742	4	0.00119	2	0.0057	4	1.06	1bp poly.
LJS	GOT1p1	6	0.02401	13	0.00195	4	NA	NA	0.74	
	GOT1p2	10	0.02296	29	0.00495	15	0.02223	29	−1.48	
	GOT1Sr	4	0.00186	1	0.00136	5	0.0008	5	−0.446	
	GOT1Sd	8	0.0163	6	0.00494*	5	0.00977	23	0.23	1bp poly.
	GOT1_6a	10	0.00211	1	0.00044	1	0.00092	0	0.12	
AB	GOT1p1	6	0.00111	1	0.0004	1	NA	NA	−1.13	
	GOT1p2	6	0.00412	3	0.00087	1	0.00399	3	0.56	
	GOT1Sr	4	0.00479	2	0.0023*	3	0.00102	4	1.06	(stop codon poly.)
	GOT1Sd	4	0	0	0.00287	2	0.00045	1	−0.754	
	GOT1_6a	10	0.00214	1	0.00043	1	0.00304	4	1.5	
SCN	GOT1p1	6	0.01356	8	0	0	NA	NA	0.27	
	GOT1p2	10	0.01774	14	0.00155	4	0.01747	14	−0.05	
	GOT1Sr	2	0	0	0	0	0.00122	6	NA	
	GOT1Sd	9	0.036	8	0.0123*	9	0.00632	9	0.672	4bp poly., 1bp poly., 4bp fixed
	GOT1_6a	8	0.016	11	0.0023	5	0.0165	14	−0.21	

# Hap. indicates the number of haplotypes sequenced for each gene. S indicates the number of segregating sites for each type of polymorphism (syn-synonymous, non-syn-nonsynonymous, syn+nc-noncoding and synonymous). π gives the average pairwise sequence divergence for each of the same three catergories of sites. Taj. D is the value of Tajima’s D. Poly. indicates that site is polymorphic.

*Note calculations of non-synonymous polymorphism correct reading frame caused by indels in coding region for comparison to other sequences.

Although there is evidence for inter-paralog gene conversion for the *GOT1Sd* and *GOT1Sr* paralogs as well, it does not appear to have been substantial enough to result in both copies retaining their open reading frames in all haplotypes. For the SD, LJS, and particularly SCN populations there are fixed and polymorphic indels in exons in *GOT1Sd* that should disrupt the reading frame and result in greatly truncated mRNAs (Table 2). In the AB population there appears to be a premature stop codon in the *GOT1Sr* that is polymorphic in this population. For both the *GOT1Sr* and *GOT1Sd* paralogs elevated k_a_/k_s_ ratios are seen for some comparisons further suggesting reduced functional constraint (Table 1). For the *GOT1p2* gene one haplotype in the SD population also had an insertion that would disrupt the reading frame suggesting that non-functional alleles can also be found at this locus. A one bp deletion was found in the coding region for a single haplotype in *GOT1*_*6a* in the SD population as well. Only for the *GOT1p1* gene copy were no such truncating or frameshift polymorphisms found in any of this set of four populations of *T*. *californicus* for these five GOT1 homologs.

## Discussion

I have identified a set of homologous genes from *T*. *californicus* that appear to encode aspartate transaminase proteins and these genes display a number of interesting patterns of inter-locus gene conversion. In discussing these results, first, I will discuss the potential deeper level relationships among these duplicates within and between species and then, second, I will look at the interesting patterns of gene conversion in two pairs of more closely related duplicates.

The cytosolic GOT1 proteins have undergone a number of gene duplication events in copepods and in the *T*. *californicus* lineage. The *GOT1p1*/*GOT1p2* paralogs cluster phylogenetically with cytosolic GOT1 proteins in other species of arthropods and are their most likely orthologs. The relationships of the other three *GOT1* paralogs to other GOT1 proteins are not resolved with the exception of a weakly supported relationship to putative GOT1 paralogs in two other distantly related copepod species (*Caligus clemensi* and *Lepeoophtheirus salmonis*). The lack of deeply divergent *GOT1* paralogs in other sequenced metazoan genomes suggests that the duplication events producing the *GOT1*_*6a* and *GOT1Sd*/*GOT1Sr* paralogs may have occurred within copepods and were not the result of an ancient metazoan duplication event. Other examples of older duplicates of aspartate transaminases in animals are restricted to individual clades such as mammals as can be seen in panther gene family trees http://www.pantherdb.org/ for aspartate aminotransferases [27]. If the duplications did occur within copepods, perhaps relatively high levels of amino acid divergence in these paralogs are obscuring their relationship to the other GOT1 proteins. Regardless of the deeper level relationships, it is clear that the duplications that have resulted in the production of the *GOT1Sd*/*GOT1Sr* and *GOT1p1*/*GOT1p2* gene pairs occurred more recently than these deeper splits. Most likely these splits occurred in the common ancestor of these four populations of *T*. *californicus* given the presence of each copy in each population.

The *GOT1p1* paralog is the most conserved of the five paralogs with no evidence for segregating non-functional alleles (Table 2) and it has the highest levels of constraint as measured by k_a_/k_s_ values (Table 1). The higher expression level of the *GOT1p1* copy, coupled with potential matches between predicted amino acid differences and allozyme allele differences among populations together suggest that the *GOT1p1* paralog could be the same locus as the *GOT1* allozyme used previously to examine genetic variation among *T*. *californicus* populations [19,28,29] and may be the primary cytosolic aspartate transaminase protein in this species. The *GOT1p2* paralog has slightly lower levels of constraint than the *GOT1p1* paralog and has one haplotype that contains a frameshift polymorphism in this sample of sequences from the SD population. Of the five paralogs, the *GOT1Sd* gene is behaving the most like a pseudogene. It does not appear to be expressed at detectable levels and has a series of frameshift substitutions in each of the populations that disrupt the reading frame (with the exception of the AB population).

Turning now to the patterns of gene conversion in the more recently duplicated pairs of paralogs, *GOT1Sd*/*GOT1Sr* and *GOT1p1*/*GOT1p2*, it is clear that there has been gene conversion in the past within each pair. There is no evidence of gene conversion between the more divergent paralogs, e.g. between *GOT1Sr* and *GOT1*_*6a*. There are numerous likely gene conversion tracks resulting from both inter- and intra-locus events between pairs for both of these sets of paralogs (Figures 1 and 2; Additional file 3: Table S2). For the *GOT1Sd*/*GOT1Sr* pair the inter-paralog gene conversion events are largely restricted to the exonic sequences with a large intron becoming largely un-alignable between paralogs. The *GOT1Sd* gene appears to be evolving as a pseudogene in several populations as discussed above despite evidence for inter-locus gene conversion events with the largely intact *GOT1Sr* gene. Apparently these gene conversion events are not happening frequently enough to maintain the open reading frame of this *GOT1Sd* copy in all populations. In contrast to the *GOT1Sd*/*GOT1Sr* pair of genes, there are no introns in the coding sequences of the *GOT1p1*/*GOT1p2* paralogs and the regions of elevated divergence between the two paralogs are therefore located within the single exon. Close physical proximity in the genome can facilitate interlocus gene conversion [30] and in fact, the *GOT1p1*/*GOT1p2* paralogs are tightly linked (and are also located on the same chromosome as *GOT2*; Willett CS, unpublished data). The allozyme loci *GOT1* and *GOT2* were previously shown to be linked [31], lending further credence to the idea that the *GOT1p1* and/or *GOT1p2* loci might encode the allozyme marker *GOT1* that has been previously characterized in this species.

Both pairs of paralogs *GOT1Sd*/*GOT1Sr* and *GOT1p1*/*GOT1p2* show islands of genetic divergence amid regions of higher similarity but the evolutionary explanation for this pattern may differ between the two sets of duplicates. For the *GOT1Sd*/*GOT1Sr* pair the divergence is restricted to the introns and may be a result of the accumulation of substitutions that can terminate inter-paralog gene conversion in those stretches of the gene. Divergence in sequence similarity that lowers the level of gene conversion could accumulate either via the gradual accumulation of single-base differences or more rapidly by larger changes such as large indels [32,33]. The *GOT1Sd*/*GOT1Sr* paralogs have both very large size differences and low sequence similarity in the intron so that either mode of divergence could have contributed to the absence of gene conversion in these regions. Even small regions of clustered sequence divergence (with multiple substitutions or indels) can dramatically reduce the rate of gene conversion for a region of a gene [34,35]. The net result of this divergence for the *GOT1Sd*/*GOT1Sr* paralogs is that interlocus gene conversion is not likely to occur in this intronic region of the gene and these regions are free to accumulate further differences.

In contrast for the *GOT1p1*/*GOT1p2* paralogs the regions of genetic divergence occur in the exons and there are no fixed indels in these regions that could disrupt interlocus gene conversion. Teshima and Innan [7] have suggested that such regions of differentiation in the face of on-going gene conversion can be a signal that selection is maintaining divergence in the paralogs (i.e. the paralogs have begun the process of neofunctionalization). Under such a model the width of the divergent region should extend less than the average length of a gene conversion tract from the selected site or sites. A number of duplicated genes show such islands of divergence that are associated with clear functional differences in the resulting proteins (e.g. RH factor and opsin proteins [36]). Other duplicated genes in yeast and *Drosophila* show a similar pattern consistent with selection but lack evidence for functional differences [8,37]. For the *GOT1p1*/*GOT1p2* paralogs, one potential neutral explanation for this pattern could posit that gene conversion initiation is lower in these regions and that these regions have accumulated enough differentiation to begin to suppress gene conversion. An argument against this limited initiation idea is that intralocus gene conversion is common in the region of sequence differentiation between these two paralogs in the first half of the gene. This observation suggests that sequence factors are not completely suppressing the initiation of gene conversion events in the divergent regions of the gene. Other factors that could also suppress interlocus gene conversion such as indel differences are also absent. The loss of fixed divergences between paralogs for one of these islands of genetic divergence in the SD and LJS populations in the second half of the gene (discussed further below) also argues that gene conversion is still possible for these regions. Although these results are suggestive of a selective explanation, further study attempting to identify functional differences between the *GOT1p1*/*GOT1p2* paralogs is needed to confirm or reject this hypothesis.

A region of high polymorphism and lowered divergence between a set of alleles in the *GOT1p1*/*GOT1p2* paralogs in the SD and LJS populations is likely to have been created by inter-paralog gene conversion. The patterns of variation and phylogenetic evidence (Figure 1 and Figure 4) are consistent with one-way transfers of variation from each population’s *GOT1p1* locus to the *GOT1p2* locus. One-way exchange like this is consistent with other studies where gene conversion shows biased directionality [2,38]. The net result of this directional gene conversion is to transfer variants from one paralog to the other. In this case this transfer is limited to the second half of the gene resulting in haplotypes that are a chimera of the *GOT1p1* and the *GOT1p2* paralogs and this transfer also results in an increase in the levels of polymorphism in this region of the gene. The chimeric protein that results is substantially altered from that produced by other *GOTp2* alleles, differing by 8 amino acids, while still differing from *GOTp1* by 20 amino acid in the first half of the gene.

It is possible that gene conversion events that result in greatly augmented polymorphism in gene duplicates are effectively neutral, but in a number of other cases they appear to be under selection, often occurring in genes undergoing selection for diversification [11-17,39]. For the *GOT1p1* and *GOT1p2* genes there is not a clear signal of diversifying selection in comparisons of orthologous copies across populations with K_a_/K_s_ values much lower than one (Table 2). Without any further functional information it is difficult to say whether the gene conversion events that resulted in greatly increased diversity in the *GOT1p2* gene in the SD and LJS population are adaptive in nature. Clearly this process has generated a large amount of novel variation at this locus both in DNA and protein sequence.

## Conclusions

The two sets of duplicate genes of *GOT1* illustrate different patterns of evolution with ongoing gene conversion among duplicated copies. The set of *GOT1Sd*/*GOT1Sr* genes appear to be in the process of diverging with gradually decreasing gene conversion given that one copy does not maintain its open reading frame and does not appear to be expressed. The central intron in this gene is already quite divergent. In contrast for the *GOT1p1*/*GOT1p2* pair, gene conversion is maintaining much higher similarity in some regions of the gene but other exonic portions are substantially diverged. The combination of these islands of genetic divergence between paralogs with rare gene conversion events has the ability to construct radically different haplotypes from the combination of variation in both paralogs (as has happened in the SD and LJS *GOT1p2* gene). Further work on the function of these two duplicates could help to determine whether there are likely to be adaptive differences between these copies.

## Methods

### Isolation and sequencing of GOT1 homologs

The putative *GOT1* homologs were uncovered from *T*. *californicus* using an analogous strategy to that used to obtain the *GOT2* homolog in this species [24]. Briefly, a cDNA library was screened for putative homologs using a PCR-RACE procedure with primers designed to match conserved regions of GOT proteins from a range of species. Five homologs of GOT1 were eventually identified using this screen after cloning and sequencing the products to separate the more closely related paralogs. Initial work was done for the San Diego population in southern California (SD, 32.7457˚N, 117.2550˚W, San Diego County, CA). Three other sites were used to examine the evolution of these *GOT1* paralogs, two more in southern California, La Jolla (LJS, 32.8434˚N, 117.2808˚W, San Diego County, CA), and Abalone Cove (AB, 33.7377˚N, 118.3753˚W, Los Angeles County, CA), and one site in central California, Santa Cruz (SCN, 36.9495˚N, 122.0470˚W, Santa Cruz County, CA). These sites were selected because they capture a number of divergent lineages of *T*. *californicus* and have been used extensively in other studies of sequence evolution in this species [22,24,26].

To obtain the sequences of each of these five *GOT1* paralogs, DNA from single copepods was obtained using a proteinase-K cell-lysis method [40]. Table 3 lists the primers that were used in PCR reactions that can specifically amplify each paralogous sequence under the specified set of conditions. PCR products were directly sequenced using capillary sequencing. Between two and ten haplotypes were sequenced for each gene from each of the four populations (the numbers of sequenced haplotypes are given in Table 2). To verify the sequence of the expressed mRNA for these paralogs, RNA from single copepods was isolated using the TRI reagent RNA isolation procedure (Sigma Chemical, Saint Louis, MO). After making cDNA from these preparations, the mRNA sequence was obtained from individual copepods from each of the four populations listed above for the two GOT1p1/2 paralogs and the *GOT1Sr* gene (there did not appear to be any product from the *GOT1Sd* gene in these populations).

**Table 3:** **Primer sequences and amplification conditions for GOT1 paralogs from *T***. ***californicus***

**Gene Region**	**Forward Primer (5′ to 3′)**	**Reverse Primer (5′ to 3′)**	**Size (bp)**	**Ann. Temp.**
GOT1p1	AGAAGTTGGTCATTCATTCTTCATC	CTTATTGACGGCCTCATTGATGGA	1243	58°C
GOT1p2	ATATCCGTGCCCAAAAGCCTAC	CTTATTGACGGCCTCATTGATGGA	1249	58°C
GOT1Sr (5′end)	AGTATCCCAACATGTCTGCCTTCG	GCAATATAGATAATGAATAACGACACT	~1700^1^	63˚C^2^
GOT1Sr (middle)	GAGTGTCGTTATTCATTATCTATATTGC	TCCTCAACCAGGTTTTAGAGTCA	1751 SD, 1722 AB, 2855 SCN	63°C
GOT1Sr (3′end)	CAATTGACTCTAAAACCTGGTTGAG	GGTTATTCTTGAATTTGTTGTGCTTCGT	~1400bp	63°C
GOT1Sd	CAGGAACAATGGAAGATCATAGCCA	GGTTATTCTTGAATTTGTTGTGCTTCGT	~1700bp	
GOT1_6A	ATGGCCACAACCAAGTTTATTG	TGGTCTTTGATGGGGCCTTCGTTCG	~1600bp	55°C

^1^ There are small size variations due to indels in these fragments.

^2^ For the amplifications of the GOT1Sr gene fragments the Phusion kit (NEB) was used to amplify products.

### Sequence analyses

All sequences were aligned and edited using Sequencer v4.8 software (Genecodes, Ann Arbor, Michigan). The program DNAsp v.5 [41] was used to perform the polymorphism and divergence analyses for each gene. In addition to calculations of polymorphism and divergence (including analyses over sliding windows), Tajima’s D test [42] was also implemented. The program GENECONV (version 1.81a http://www.math.wustl.edu/~sawyer/geneconv/) was used to identify regions of the paralogous genes that have sequence patterns consistent with gene conversion [25]. Gene conversion events were identified both within and between paralogs within a single population by setting up the group structure within the file and allowing only gene conversion events within populations. The protein variability server (http://imed.med.ucm.es/PVS/) was used to look at patterns of amino acid conservation across GOT1 proteins of arthropods [43]. Conservation was measured by looking at the diversity of amino acids at each site using the Shannon entropy H value.

Phylogenetic trees were constructed using both parsimony and Bayesian analyses with amino acid sequence data and only with parsimony for DNA sequence data from within *Tigriopus*. The program PAUP*v4b10-×86 [44] was used for the parsimony reconstructions of relationships among *GOT1p1*/*GOT1p2* haplotypes. Heuristic searches were done with 100 random starting trees using either the first 923 bp of the sequence or the last 282 bp in separate analyses. A similar search approach was used for analyses of the divergent sets of GOT amino acid sequences for parsimony analyses. A variety of search conditions using Bayesian analyses and the program MrBayes v3.1.2 [45] were also performed on these protein alignments but did not provide strong support for unresolved relationships in the parsimony analyses.

## Availability of supporting data

Sequences are available in Genbank with the accession numbers [KF135593 to KF135616]. The data sets (sequence alignments) supporting the results of this article are available in the Dryad repository http://dx.doi.org/10.5061/dryad.8r6jp.

## Competing interests

The author declares no competing interests, financial or otherwise, regarding this manuscript.

## Supplementary Material

Additional file 1: Figure S1 Most parsimonious tree for relationships among GOT paralogs. Click here for file

Additional file 2: Table S1 Genetic divergence among orthologs and paralogs of GOT1 in *T*. *californicus*. Numbers of fixed substitutions and sites are for comparisons of GOT1 homologs. Click here for file

Additional file 3: Table S2 List of potential sites of gene conversion. Results are obtained from the program GENE_CONV. Click here for file

Additional file 4: Figure S2 Plot of conservation of GOT1 proteins from arthropods with divergent regions of *T*. *californicus GOT1p1*/*GOT1p2* highlighted. Click here for file
