# Supplementary material for: Gene conversion yields novel gene combinations in paralogs of GOT1 in the copepod Tigriopus californicus
Source: BMC Evol Biol. 2013 Jul 12;13:148. doi: 10.1186/1471-2148-13-148 (PMC3728101; doi:10.1186/1471-2148-13-148)
Supplement: Additional file 1: Figure S1 — Most parsimonious tree for relationships among GOT paralogs. [file 1471-2148-13-148-S1.pdf]

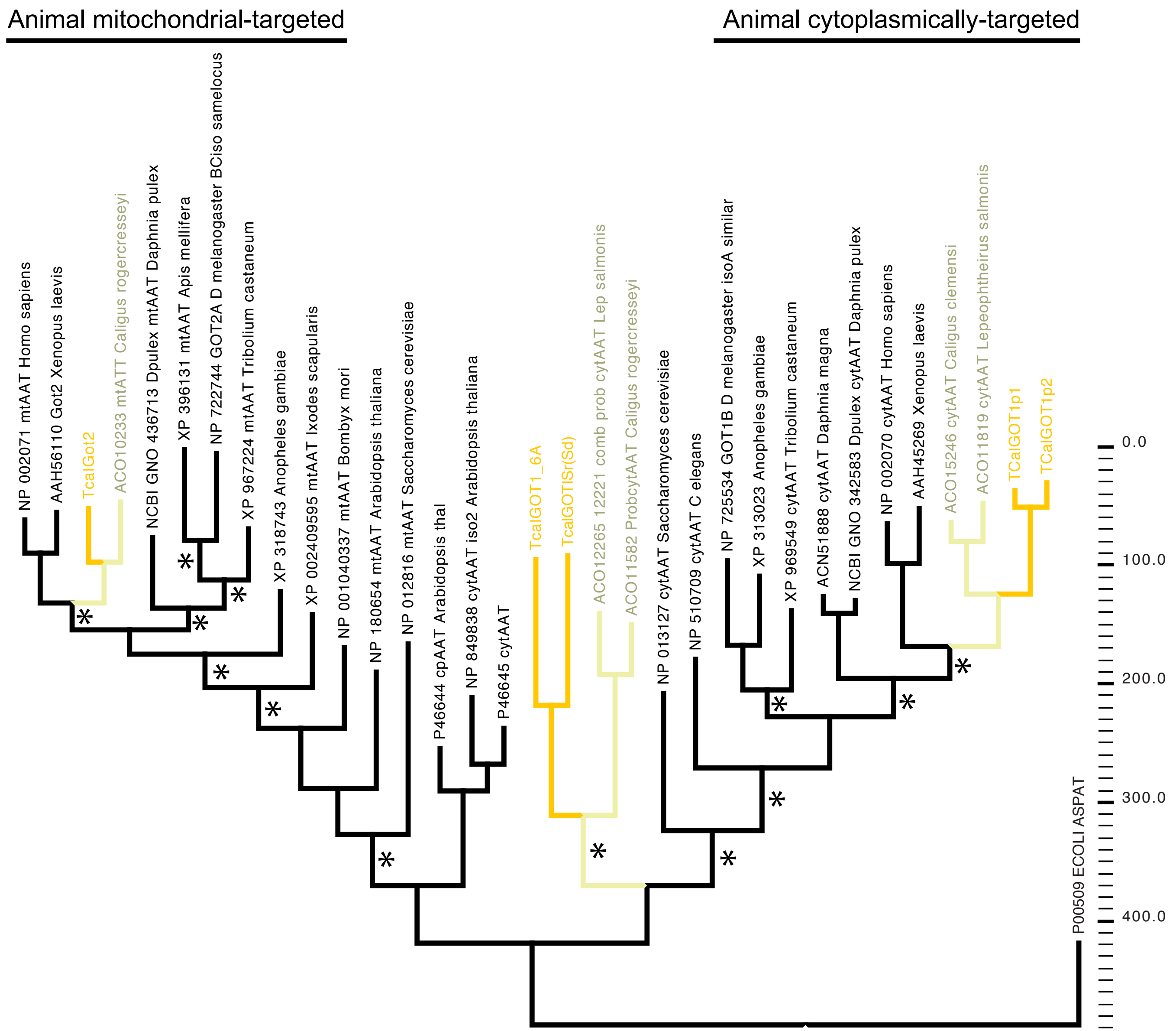

Supplemental Figure 1. Most parsimonious tree for relationships among GOT paralogs. Copepod proteins from *T. californicus, Caligus clemensi,* and *Lepeophtheirus salmonis* are highlighted. Many of the basal branches in tree have little support with bootstrap values lower than 50 (indicated by an *). The GOT1p1/2 proteins fall out with strong support with other animal GOT1 proteins but the GOT1Sd/r and GOT1_6A protein positions are not well-supported within the GOT1 proteins. Analogous results were found for Bayesian analyses using a number of different parameter settings with the program MrBayes. Note: the partial GOT1Sd sequence was not included in this analysis but partial analyses confirm it is very closely related to GOT1Sr (3 percent amino acid divergence). Tree is rooted with Ecoli ASPAT protein. Tree was constructed using the program PAUP* with heuristic searches and multiple random starting trees.
