# Supplementary material for: Gene conversion yields novel gene combinations in paralogs of GOT1 in the copepod Tigriopus californicus
Source: BMC Evol Biol. 2013 Jul 12;13:148. doi: 10.1186/1471-2148-13-148 (PMC3728101; doi:10.1186/1471-2148-13-148)
Supplement: Additional file 2: Table S1 — Genetic divergence among orthologs and paralogs of GOT1 in T. californicus. Numbers of fixed substitutions and sites are for comparisons of GOT1 homologs. [file 1471-2148-13-148-S2.pdf]

Supplemental Table 1. Genetic divergence among orthologs and paralogs of GOT1 in *T. californicus.*

	Between paralogs				GOT1p1 orthologs			GOT1p2 orthologs		
	SD p1/p2	LJS p1/p2	AB p1/p2	SCN p1/p2	SD p1/LJS p1	SD p1/AB p1	SD p1/SCN p1	SD p2/LJS p2	SD p2/AB p2	SD p2/SCN p2
#syn	54	57	77	77	0	12	21	0	13	16
#syn sites	265.8	273.1	273	272.9	272	271	271	267	267	267
ks	0.237	0.245	0.354	0.354	0	0.046	0.082	0	0.050	0.062
#non-syn	22	22	29	30	0	2	3	0	5	3
#ns sites	901.2	917.9	918	918.1	922	919.9	920.1	900.3	900	900
ka	0.025	0.024	0.032	0.033	0	0.002	0.003	0	0.006	0.003
ka/ks	0.105	0.100	0.091	0.094	-	0.048	0.040	-	0.111	0.053

	Between paralogs				GOT1Sd orthologs			GOT1Sr orthologs		
	SD Sd/Sr	LJS Sd/Sr	AB Sd/Sr	SCN Sd/Sr	SD Sd/LJS Sd	SD Sd/ AB Sd	SD Sd/ SCN Sd	SD Sr/LJS Sr	SD Sr/AB Sr	SD Sr/SCN Sr
#syn	4	2	1	4	1	1	5	1	7	14
#syn sites	119.7	127.9	108	114.7	119.2	105.4	108	267	267.5	278.4
ks	0.034	0.016	0.009	0.036	0.008	0.010	0.048	0.004	0.027	0.052
#non-syn	4	1	5	6	1	6	7	2	15	16
#ns sites	384.3	409.1	348	368.3	381.8	344.6	354	855	872.5	879.6
ka	0.010	0.002	0.015	0.016	0.003	0.018	0.020	0.002	0.017	0.018
ka/ks	0.307	0.155	1.557	0.461	0.311	1.845	0.419	0.624	0.653	0.354

	GOT1_6a orthologs					
	SD 6a/LJS 6a	SD 6a/AB 6a	SD 6a/SCN 6a	LJS 6a/AB 6a	LJS 6a/SCN 6a	AB 6a/SCN 6a
#syn	1	8	10	11	14	7
#syn sites	252.7	255.5	256	251.9	252.4	258.8
ks	0.004	0.032	0.040	0.045	0.058	0.028
#non-syn	1	2	9	3	10	9
#ns sites	797.4	809.6	809	798.1	797.7	824.2
ka	0.001	0.002	0.011	0.004	0.013	0.011
ka/ks	0.316	0.077	0.279	0.084	0.219	0.399

Divegence between copies are computed for the coding region for synonymous sites (syn) and nonsynonymous sites (non-syn). Rates per site are ks and ks respectively and are calculated by applying a Jukes-Cantor correction to raw divergences.
