# Supplementary material for: Gene conversion yields novel gene combinations in paralogs of GOT1 in the copepod Tigriopus californicus
Source: BMC Evol Biol. 2013 Jul 12;13:148. doi: 10.1186/1471-2148-13-148 (PMC3728101; doi:10.1186/1471-2148-13-148)
Supplement: Additional file 3: Table S2 — List of potential sites of gene conversion. Results are obtained from the program GENE_CONV. [file 1471-2148-13-148-S3.pdf]

Supplemental Table 2. List of potential sites of gene conversion. Results are obtained from the program GENE_CONV.

Potential novel events GOT1p1/2	event involving SD GOT1p2 paralog	event involving SD GOT1p2 paralog	involving SD GOT1p1 paralog	Sim P value	BC KA P value	Begin	End	Length	Num Poly	Num Dif	Total Diffs
1a	GOT1p2_SDm11a	GOT1p2_sd2b		0.0289	> 1.0	1	605	605	122	0	13
1a	GOT1p2_SDm11b_insert	GOT1p2_SDm10a_p1like		0.0289	> 1.0	1	605	605	122	0	13
1b	GOT1p2_sd2a, GOT1p2_SDm10a_p1like	GOT1p2_SD1b_a_2lk, GOT1p2_SDm9_2lk		0.0001	0.0018	139	605	467	96	0	30
1b	GOT1p2_SDm11a	GOT1p2_SDm9_2lk, GOT1p2_SD1b_a_2lk		0	0.0004	139	605	467	96	0	33
1c	GOT1p2_SD1b_a_2lk, GOT1p2_SDm9_2lk	GOT1p2_sd2b		0	0.0012	139	647	509	102	0	29
1d	GOT1p2_SDm11b_insert	GOT1p2_SDm9_2lk, GOT1p2_SD1b_a_2lk		0	4E-05	139	713	575	108	0	33
1e	GOT1p2_SDm10b_p1like	GOT1p2_SD1b_a_2lk, GOT1p2_SDm9_2lk		0.0035	0.2061	285	570	286	65	0	31
1f	GOT1p2_SDm9_2lk, GOT1p2_SD1b_a_2lk,	GOT1p2_SD1b_B_1lk, GOT1p2_SDm9a_1lk		0.0032	0.1805	313	550	238	55	0	37
2a	GOT1p2_SDm9_2lk, GOT1p2_SD1b_a_2lk		GOT1p1_SDm9b, GOT1p1_sd1b_A_B, GOT1p1_SDm9a, GOT1p1_sd2_A, GOT1p1_sd2_B	0.0001	0.0027	544	713	170	20	0	112
2b	GOT1p2_SDm11b_insert		GOT1p1_sd2_B, GOT1p1_SDm9b, GOT1p1_SDm9a, GOT1p1_sd2_A	0	0.0008	544	770	227	30	0	88
2c	GOT1p2_SDm11b_insert		GOT1p1_sd1b_A_B	0	0.0006	544	788	245	31	0	87
3a	GOT1p2_SDm10b_p1like		GOT1p1_SDm9b, GOT1p1_sd1b_A_B, GOT1p1_SDm9a, GOT1p1_sd2_A	0	0.0003	897	1001	105	30	0	92
3b	GOT1p2_sd2b, GOT1p2_SDm10a_p1like		GOT1p1_SDm9b, GOT1p1_sd1b_A_B, GOT1p1_SDm9a, GOT1p1_sd2_A	0	0.0003	905	1030	126	31	0	90
3c	GOT1p2_SD1b_B_1lk, GOT1p2_SDm11a, GOT1p2_SDm11b_insert, GOT1p2_SDm9a_1lk		GOT1p1_SDm9b, GOT1p1_sd1b_A_B, GOT1p1_SDm9a, GOT1p1_sd2_A	0	0.0002	919	1030	112	29	0	96
3d	GOT1p2_sd2a		GOT1p1_SDm9b, GOT1p1_sd1b_A_B, GOT1p1_SDm9a, GOT1p1_sd2_A	0	2E-05	919	1094	176	33	0	94
3e	GOT1p2_SDm10b_p1like		GOT1p1_sd2_B	0.0369	> 1.0	946	1001	56	16	0	92
3f	GOT1p2_SD1b_B_1lk, GOT1p2_SDm11a, GOT1p2_SDm11b_insert, GOT1p2_SDm9a_1lk		GOT1p1_sd2_B	0.0075	0.3818	946	1030	85	19	0	90
3g	GOT1p2_sd2a		GOT1p1_sd2_B	0.0003	0.0148	946	1094	149	23	0	94
	<u>event involving LJS GOT1p2 paralog</u>	<u>event involving LJS GOT1p2 paralog</u>	<u>involving LJS GOT1p1 paralog</u>								
1a	GOT1p2_LJSm19b_p1like	GOT1p2_LJSm18b, GOT1p2_LJSm20a, GOT1p2_LJSm20b		0	2E-05	1	713	713	134	0	28
1b	GOT1p2_LJSm19b_p1like	GOT1p2_LJSm17a, GOT1p2_LJSm17b		0.0021	0.1224	196	446	251	62	0	34
1c	GOT1p2_LJSm19b_p1like	GOT1p2_LJSm18a, GOT1p2_LJSm21a,GOT1p2_LJSm19a		0	0.0003	196	713	518	101	0	32
2a	GOT1p2_LJSm21a, GOT1p2_LJSm19a, GOT1p2_LJSm17b, GOT1p2_LJSm18a, GOT1p2_LJSm20a, GOT1p2_LJSm20b		GOT1p1_LJSm19a, GOT1p1_LJSm19b	0.007	0.3474	544	647	104	14	0	113
2b	GOT1p2_LJSm21a, GOT1p2_LJSm19a, GOT1p2_LJSm17b, GOT1p2_LJSm18a, GOT1p2_LJSm20a, GOT1p2_LJSm20b		GOT1p1_LJSm18b, GOT1p1_LJSm18a, GOT1p1_LJSm17a	0.0001	0.0017	544	713	170	20	0	114
2c	GOT1p2_LJSm19b_p1like		GOT1p1_LJSm18a, GOT1p1_LJSm17a, GOT1p1_LJSm18b	0	0.0013	544	761	218	28	0	91
2d	GOT1p2_LJSm21b		GOT1p1_LJSm17a, GOT1p1_LJSm18a, GOT1p1_LJSm18b	0.0105	0.5206	551	647	97	13	0	116
2e	GOT1p2_LJSm21b		GOT1p1_LJSm19a, GOT1p1_LJSm19b	0.001	0.037	551	671	121	16	0	116
3a	GOT1p2_LJSm19b_p1like		GOT1p1_LJSm17b	0.0067	0.3236	649	845	197	19	0	91
3b	GOT1p2_LJSm19b_p1like		GOT1p1_LJSm19b	0	0	649	1030	382	57	0	86
4a	GOT1p2_LJSm21a, GOT1p2_LJSm19a, GOT1p2_LJSm17a, GOT1p2_LJSm17b, GOT1p2_LJSm18a, GOT1p2_LJSm20b, GOT1p2_LJSm20a, GOT1p2_LJSm18b		GOT1p1_LJSm18a, GOT1p1_LJSm18b, GOT1p1_LJSm19a	0.0001	0.0063	715	903	189	19	0	112
4b	GOT1p2_LJSm21a, GOT1p2_LJSm19a, GOT1p2_LJSm17a, GOT1p2_LJSm17b, GOT1p2_LJSm18a, GOT1p2_LJSm20b, GOT1p2_LJSm20a, GOT1p2_LJSm18b		GOT1p1_LJSm17a	0	0.0015	715	917	203	21	0	111
3c	GOT1p2_LJSm19b_p1like		GOT1p1_LJSm17a	0	0	790	1030	241	40	0	88
3d	GOT1p2_LJSm19b_p1like		GOT1p1_LJSm17b	0	2E-05	847	1013	167	35	0	91
3e	GOT1p2_LJSm19b_p1like		GOT1p1_LJSm18a, GOT1p1_LJSm19a, GOT1p1_LJSm18b	0	0.0007	919	1030	112	29	0	91
	<u>event involving AB GOT1p2 paralog</u>		<u>involving AB GOT1p1 paralog</u>								
1	GOT1p2_AB_1b_B, GOT1p2_AB_1b_A, GOT1p2_ABm10b, GOT1p2_ABm10a		GOT1p1_ABm10a, GOT1p1_AB1b_A, GOT1p1_ABm10b, GOT1p1_AB1b_B	0.0013	0.0766	75	231	157	16	0	112
2a	GOT1p2_AB_1b_B, GOT1p2_AB_1b_A, GOT1p2_ABm10a, GOT1p2_ABm10b		GOT1p1_ABm10a, GOT1p1_AB1b_A, GOT1p1_ABm13a_b, GOT1p1_ABm10b, GOT1p1_AB1b_B	0	0	515	744	230	28	0	112
2b	GOT1p2_ABm11a_b		GOT1p1_ABm10a, GOT1p1_AB1b_A, GOT1p1_ABm13a_b, GOT1p1_ABm10b, GOT1p1_AB1b_B	0	0	515	767	253	31	0	111
	<u>event involving SCN GOT1p2 paralog</u>		<u>involving SCN GOT1p1 paralog</u>								
1a	GOT1p2_SC3b, GOT1p2_SCN1a_b, GOT1p2_SCN4a_b, GOT1p2_SCN5a, GOT1p2_SCN5b, GOT1p2_SCN6b		GOT1p1_SC1a, GOT1p1_SCN4a_b, GOT1p1_SCN3a, GOT1p1_SC1b, GOT1p1_SC1a, GOT1p1_SCN3b	0.0428	> 1.0	590	683	94	11	0	119
1b	GOT1p2_SC3a, GOT1p2_SCN6a		GOT1p1_SCN4a_b, GOT1p1_SCN3a, GOT1p1_SC1b	0.0013	0.075	590	725	136	15	0	117

Potential novel events GOT1Sr/d	event involving SD GOT1Sr paralog	event involving SD GOT1Sd paralog	event involving SD GOT1Sd paralog	Sim P value	BC KA P value	Begin	End	Length	Num Poly	Num Dif	Total Diffs
1	all SD GOT1Sr		all SD GOT1sd	0.0066	0.3153	67	147	81	17	0	167
2a		SD3f_1Sa,b_full	GOT1S_sd8fb_full	0.0011	0.0904	554	1318	765	230	0	15
2a		sd4f_1Sa,b_full, SD_6f_1Sa,b_Full, SD_7f_1Sa, b_full	SD3f_1Sa,b_full	0.0025	0.1722	554	1318	765	230	0	14
2b		sd4f_1Sa,b_full, SD_6f_1Sa,b_Full, SD_7f_1Sa, b_full	GOT1S_sd8fa_full	0.0221	0.9915	554	1408	855	234	0	11
2b		GOT1S_sd8fa_full	GOT1S_sd8fb_full	0.0221	0.9915	554	1408	855	234	0	11
3	all SD GOT1Sr		all SD GOT1sd	0	0	911	1148	238	52	0	167
	<u>event involving LJS GOT1Sr paralog</u>	<u>event involving LJS GOT1Sd paralog</u>	<u>event involving LJS GOT1Sd paralog</u>								
1	all LJS GOT1Sr		LJS_m17b_hdel, LJS_m20b_hdel	0	1E-05	67	208	142	37	0	153
2a		LJS_m17b_hdel	LJS_m17a_hdel, LJS_m18a,b_del, GOT1S_LJS_m19a, b_del, LJS_m20a_hdel	0	9E-05	411	915	505	200	0	29
2b		LJS_m20b_hdel	LJS_m17a_hdel, LJS_m18a,b_del, GOT1S_LJS_m19a, b_del, LJS_m20a_hdel	0	0.0032	547	915	369	161	0	29
3a	all LJS GOT1Sr		LJS_m17b_hdel, LJS_m20b_hdel	0	2E-05	911	1038	128	35	0	153
3b	all LJS GOT1Sr		LJS_m18a,b_del, GOT1S_LJS_m19a,b_del, LJS_m17a_hdel, LJS_m20a_hdel	0	1E-05	979	1129	151	32	0	171
	<u>event involving AB GOT1Sr paralog</u>		<u>event involving AB GOT1Sd paralog</u>								
1	all AB GOT1Sr		all AB GOT1Sd	0	0	920	1073	154	33	0	202
2	all AB GOT1Sr		all AB GOT1Sd	0	0.0001	1129	1289	161	22	0	200
	<u>event involving SCN GOT1Sr paralog</u>	<u>event involving SCN GOT1Sd paralog</u>	<u>event involving SCN GOT1Sd paralog</u>								
1		SC4_1Sa, b, GOT1S_SCN6a	SCm1astkb_1S, b	0.0021	0.1566	67	551	485	101	0	34
2a		SCm1astka_1S	GOT1S_SCN6b	0.0076	0.3758	562	805	244	124	0	25
2b		SC4_1Sa,b, GOT1S_SCN6a	SCm1astka,b_1S	0.0003	0.0151	596	863	268	123	0	34
2b		SCm1astkb_1S	GOT1S_SCN6b	0.0092	0.498	596	805	210	112	0	27
3a	GOT1sr_SCN_1La		GOT1S_SCN6b	0.0164	0.7072	1116	1182	67	13	0	192
3a	GOT1sr_SCN_1L		GOT1S_SCN6b	0.0183	0.7692	1116	1182	67	13	0	191
3b	GOT1sr_SCN_1La		SCm1astka_1S	0.0469	> 1.0	1229	1328	100	11	0	202
3b	GOT1sr_SCN_1L		SCm1astka_1S	0.0487	> 1.0	1229	1328	100	11	0	201

Notes from GENE_CONV output: BC KA means Bonferroni-corrected KA (BLAST-like) Pvalues. Num Poly, Num Dif., and Tot Difs, are the number of polymorphic sites in the fragment, the number of mismatches within the fragment, and the total number of mismatches between two sequences respectively. Potential novel events refers to events predicted for different regions (each with a new number) and similar regions (added letter to number).
