# Supplementary material for: Gene conversion yields novel gene combinations in paralogs of GOT1 in the copepod Tigriopus californicus
Source: BMC Evol Biol. 2013 Jul 12;13:148. doi: 10.1186/1471-2148-13-148 (PMC3728101; doi:10.1186/1471-2148-13-148)
Supplement: Additional file 4: Figure S2 — Plot of conservation of GOT1 proteins from arthropods with divergent regions of T. californicus GOT1p1/GOT1p2 highlighted. [file 1471-2148-13-148-S4.pdf]

Supplemental Figure 2

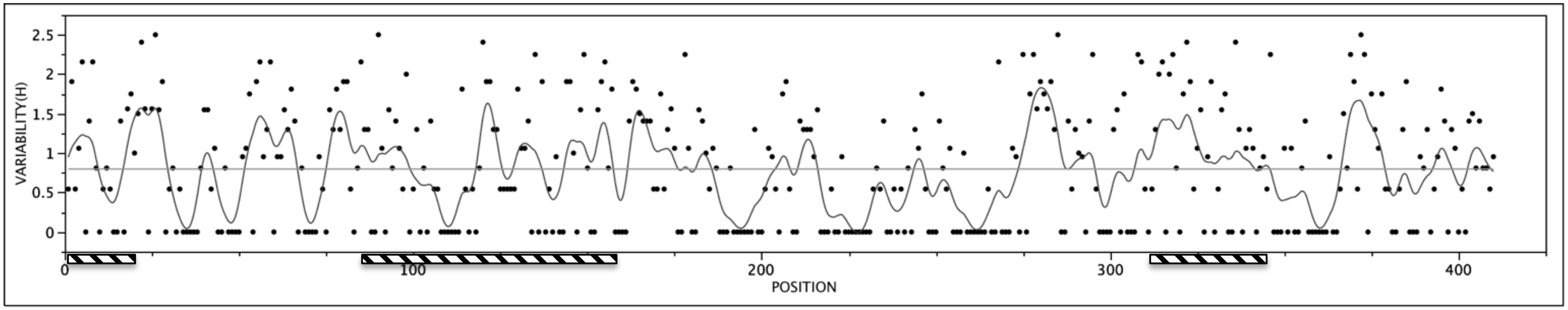

Supplemental Figure 2. Plot of conservation of GOT1 proteins from arthropods with divergent regions of *T. californicus GOT1p1/2* highlighted. The dots show the Shannon entropy H values for each amino acid position over the alignment of arthropod GOT1 proteins (with two vertebrates as well). Lower values indicate lower diversity (i.e. higher conservation). A cubic spline curve was fitted to the data with a lambda of 11 to highlight regions of higher conservation. Hatched bars under the x-axis show the regions of higher divergence between paralogs. In addition to the two *T. californicus GOT1p1/2* proteins, the following proteins were included: ACO15246 cytAAT Caligus clemensi, ACO11819 cytAAT Lepeophtheirus salmonis, ACN51888 cytAAT Daphnia magna, NP725534 GOT1B D melanogaster isoA, GNO 342583 Dpulex cytAAT Daphnia pulex, XP 313023 Anopheles gambiae,NP002070 cytAAT Homo sapiens, XP969549 cytAAT Tribolium castaneum, and AAH45269 Xenopus laevis.
